# Evone® Flow-Controlled Ventilation During Upper Airway Surgery: A Clinical Feasibility Study and Safety Assessment

**DOI:** 10.3389/fsurg.2020.00006

**Published:** 2020-02-28

**Authors:** Jeroen Meulemans, Alexander Jans, Kristien Vermeulen, Johan Vandommele, Pierre Delaere, Vincent Vander Poorten

**Affiliations:** ^1^Otorhinolaryngology, Head and Neck Surgery, University Hospitals Leuven, Leuven, Belgium; ^2^Department of Oncology, Section Head and Neck Oncology, KU Leuven, Leuven, Belgium; ^3^Anesthesiology, University Hospitals Leuven, Leuven, Belgium

**Keywords:** flow-controlled ventilation, high frequency jet ventilation, idiopathic subglottic stenosis, laryngeal cancer, laryngotracheal stenosis, transoral laser microsurgery

## Abstract

**Introduction:** During upper airway surgery in a narrowed airway due to tumor or stenosis, safe ventilation, good laryngotracheal exposure, and preservation of an adequate surgical working space are of paramount importance. This can be achieved by small-lumen ventilation such as High Frequency Jet Ventilation (HFJV). However, this technique has major drawbacks, such as air-trapping and desaturation in patients with poor pulmonary reserve. Recently, an innovative ventilating system with flow-controlled ventilation (FCV) and a small-lumen endotracheal tube, the Evone® (Ventinova, Eindhoven, The Netherlands), was introduced, claiming to counter the drawbacks of HFJV.

**Objectives:** To evaluate feasibility and safety of the Evone® FCV system in difficult upper airway surgery and to critically appraise this novel ventilation method.

**Patients and methods:** Evone® is a FCV-device using a small-bore cuffed tube (Tritube®). This ventilator actively sucks air out of the lungs, rather than relying on the passive backflow of air like in HFJV. Data related to the medical history, surgery, and anesthesia of all consecutive patients undergoing upper airway surgery with Evone® FCV ventilation were included in a tertiary center retrospective observational study.

**Results:** Fifteen Patients, with a median age of 54 years, were included. Surgical procedures and indications included laser-assisted endoscopic treatment of idiopathic subglottic stenosis (*n* = 3), tracheal stenosis (*n* = 1), and posterior glottic stenosis (*n* = 2), biopsy and/or Transoral Laser Microsurgery for laryngeal (pre)malignancy (*n* = 7) and resection of benign lesions with posterior (supra)glottic location (*n* = 2). Mean ventilation duration was 52.0 min (range 30–115 min, SD 19.6 min), mean surgery duration was 31.7 min (range 15–65 min, SD 13.2 min), mean minimal SaO_2_ was 96.3% (range 89–100%, SD 4.0%) and mean peak pCO_2_ was 41.4 mmHg (range 31–50 mmHg, SD = 5.5 mmHg). No anesthesia- or surgery-related complications, adverse events or intra-operative difficulties were reported during or after any of the 15 procedures. In all cases, compared to HFJV, Evone® FCV ventilation allowed a superior visualization and working space during the surgical procedure.

**Conclusion:** The Evone® FCV ventilation system provides excellent conditions in patients undergoing upper airway surgery, as it combines excellent accessibility and visibility of the operation site with safe and stable ventilation.

## Introduction

Despite recent advances in airway management, securing a safe airway can still be very challenging. Failure to do so is an important cause of mortality and morbidity related to general anesthesia, intensive care, and emergency situations (1, 2). For example, trying to introduce a conventional bore endotracheal tube [with an outer diameter (OD) of 10–13 mm] in a patient with a (sub)glottic or tracheal stenosis or a tumoral process in the laryngeal region may be very difficult to impossible. Another limitation of normal bore endotracheal intubation is that this thick tube, although providing good ventilation, complicates laryngeal, or tracheal surgery, as it fills a considerable part of the available space, thus impairing surgical access and impeding the surgeon's view on the operating field. These issues led to the development of specialized airway devices and techniques, such as tracheostomy, extra-corporal membrane-oxygenation, and small-lumen endotracheal tubes and their accompanying ventilation techniques (3). A well-known example of the latter is the High Frequency Jet Ventilation (HFJV). This technique relies on a non-cuffed narrow-bore endotracheal tube with an OD of around 4.3 mm and a specialized high frequency ventilator which insufflates small volumes of air at a supraphysiological rate (typically 100–150/min) through this narrow tube. Compared to a conventional endotracheal tube, the small tube used during HFJV results in superior intubation of narrowed airways and in improved surgical access, as it provides an unimpeded view of the larynx during ENT surgery. However, HFJV comes with some pitfalls and risks (4). Since a narrow tube inherently has a higher resistance than a wider tube, a lot of pressure has to be built up for airflow to develop, potentially inducing barotrauma (5). Secondly, during HFJV, the removal of air from the lungs relies completely on the passive backflow around the tube. This potentially results in the air not getting out of the lungs at the same rate as new air is being insufflated, causing hypercapnia and air trapping (6, 7). The latter causes hyperinflation, which requires stopping the ventilation, to allow the lungs of the patient to deflate. However, this, in its turn, can cause hypoxia which necessitates re-oxygenation using high oxygen tensions (FiO_2_ > 30%) and related to this interruption of an ongoing CO_2_-laser procedure due to these unsafe high oxygen tensions. This phenomenon of air trapping is more likely in patients with severe narrowing of the airway and acts as a counterweight to the benefits related to a smaller tube. Additionally, many patients who are undergoing transoral laser microsurgery (TLM) for a laryngeal cancer do have poor pulmonary reserves due to the frequent heavy smoking history, which results in frequent desaturation at the working oxygen tensions of FiO2 ≤ 30%. This also typically leads to interruptions of the surgery due to the need of ventilating at 100% oxygen, resulting in a significantly longer duration of the procedure. One last drawback of HFJV is the fact that the passive flow of air around the tube results in oscillation of the vocal cords, which can be hindering during phonosurgery or TLM (8). The most recent step in small lumen ventilation is the development of a ventilator which is able to actively remove air out of the lungs using negative pressure. Ventilators that use this novel method of active air removal and therefore do not rely on passive lung emptying are called ‘flow-controlled' ventilators (FCVs) (9). One example of such a ventilator, is the Ventrain® (Ventinova, Meerenakkerplein 7, 5652 BJ Eindhoven, The Netherlands). The negative pressure is generated using jet-flow (Bernoulli's principle) and was designed to be manually controlled. It is typically used with a cuffed small lumen tube (e.g., Tritube® by Ventinova), which optimizes the ventilation while providing airway protection, with presumably less hypercapnia and hypoxia (10, 11). Recently, this company also launched the Evone® ventilator, a flow-controlled ventilator like the Ventrain®, but automatized. This new equipment seems very promising, since it could potentially combine all benefits of other ventilation techniques, as it could provide the same stable, easy to monitor ventilation as a normal bore tube, with the benefits of increased visibility and improved working space for the surgeon related to the use of a small bore tube (3). In this observational study, we evaluate the feasibility and safety of the Evone® system and deliver a critical appraisal of this novel method of ventilation.

## Materials and Methods

### Patients

We included all consecutive patients undergoing ENT surgery with Evone® FCV ventilation instead of conventional endotracheal intubation and ventilation or HFJV, between 01-11-2017 and 01-03-2019. The decision to use Evone® was made by the chief surgeon (JM, VV) and anesthesiologist (JV, KV), based on the pathology and planned surgical procedure (e.g., laser resection of posterior glottic benign or malign lesions, treatment of severe glottis or subglottic stenosis).

### Data Collection and Statistics

Data were retrospectively retrieved from the electronic medical file. We systematically reviewed the relevant medical history of the patient, the pre-surgical anesthesia assessment, the anesthesia report, the surgical report and the postoperative notes. Fourteen Parameters [age, gender, height, weight, Body Mass Index (BMI), ASA Physical Status Classification system (ASA), Mallampati-score (MP) (12), duration of ventilation, duration of surgery, peak pCO_2_, lowest SaO_2_, pathology, surgical procedure, and complications related to the use of Evone®] were collected. Data were descriptively analyzed using SPSS version 26.0 statistical software (IBM corp., Armonk, NY, USA).

### Anesthesia and Materials

**Figure 1:**
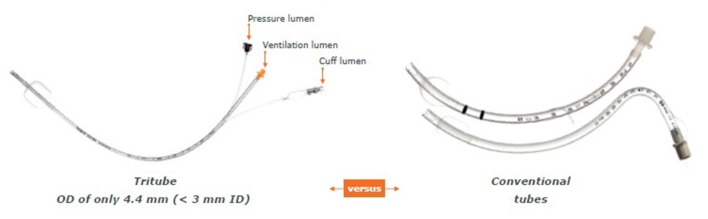
The Tritube® small-bore tube.

**Figure 2:**
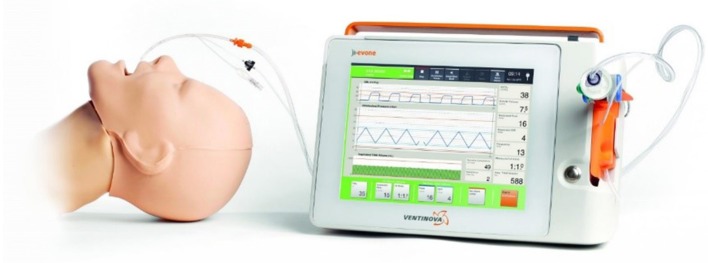
Evone® ventilator connected to the Tribute® tube.

**Figure 3:**
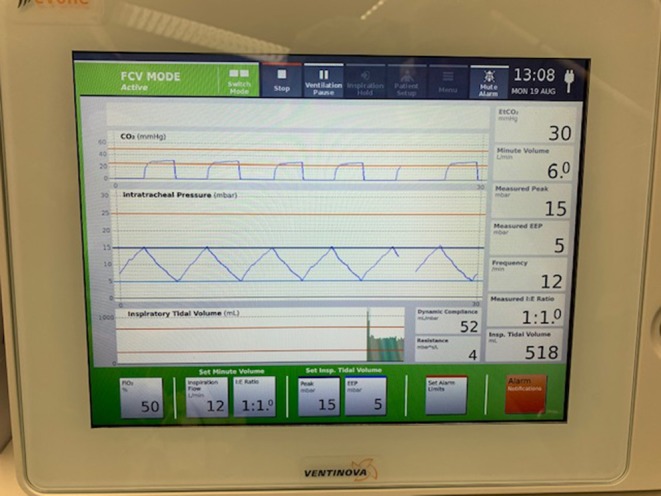
Peroperative view of the control screen of the Evone®.

One hour prior to surgery, patients were administered 0.2 mg of glycopyrronium. This long-lasting anticholinergic drug is used to reduce mucosal secretions before and during intubation to prevent the small bore intratracheal tube from getting obstructed. Next, following adequate pre-oxygenation, anesthesia was induced with intravenous propofol and remifentanil. Thereafter, neuromuscular blockade was achieved with rocuronium. Subsequently, the Tritube® endotracheal tube was advanced through the upper airway. The Tritube® (Figure 1) is a small-bore (outer diameter = 4.4 mm) tube with three lumens: one for the inflation and deflation of the cuff, one for the measurement of the pressure in the trachea, and one lumen for ventilation of the patient. The Tritube® tube is then connected to the Evone® ventilator (Figure 2). Initially, the cuff is left deflated, until full neuromuscular blockade [Train-of-four (TOF) = 0] to prevent barotrauma. After inflation of the cuff, the patient is ventilated using the FCV modus of the Evone®, which allows the anaesthesiologist to ventilate the patient with constant flows during inspiration and expiration (Figure 3).

### Technical Data and Working Mechanism Evone®

#### Flow-Controlled Ventilation

**Figure 4:**
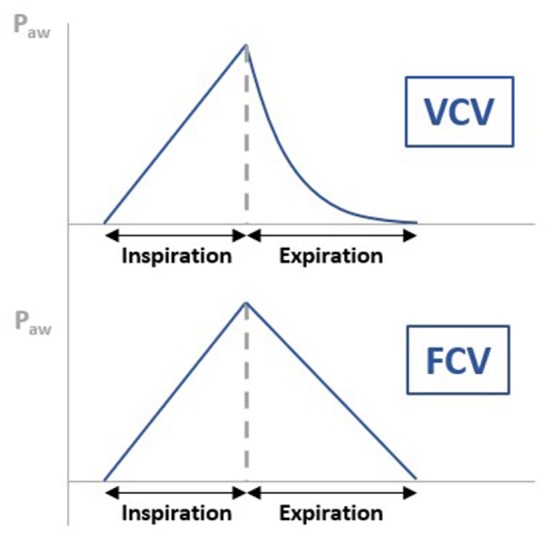
Figure illustrating the difference in airway pressure (p_aw_) curve of the conventional volume-controlled ventilation (VCV) vs. the flow-controlled ventilation (FCV) of the Evone®. In VCV, the p_aw_ decreases exponentially, giving a “bended” curve during expiration. In FCV, this part of the curve has been linearized, meaning that the flow is constant during the whole expiration.

As mentioned before, the Evone® is a flow-controlled ventilator. This means that rather than relying on the passive elasticity of the lungs to expel the air, the ventilator will produce negative pressure, actively “sucking” air out of the lungs during the expiratory phase. This difference in working mechanism can clearly be visualized when comparing the airway pressure (p_aw_) curve of the conventional volume-controlled ventilation (VCV) vs. the flow-controlled ventilation (FCV) of the Evone® (Figure 4). In VCV, the p_aw_ decreases exponentially, giving a “bended” curve during expiration. In FCV, this part of the curve has been linearized, meaning that the flow is constant during the whole expiration. This new ventilation strategy has been tested in animal models and showed improved lung recruitment and oxygenation (13).

#### Bernoulli Principle

**Figure 5:**
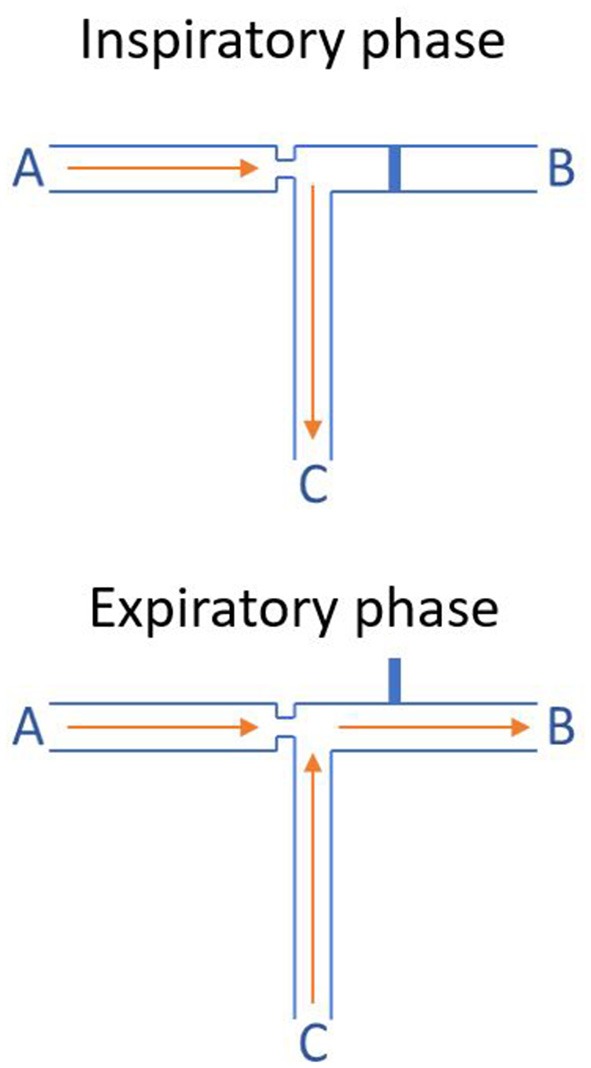
Working mechanism of the Evone® (Bernoulli principle). During the inspiratory phase, tube B, which is simply an air outlet out of the ventilator, is blocked. Therefore, the air that is forced through tube A (air inlet) is insufflated into the lungs of the patient through tube C (the endotracheal small-bore tube). During the expiratory phase, tube B is automatically opened. Therefore, the high-speed air that is insufflated through tube A shoots through tube B. This generates a low pressure, actively suctioning air out of tube C. This phenomenon relies on the Bernoulli principle and is called jet entrainment.

Thanks to the Bernoulli principle, the Evone® is able to produce a negative pressure. This is achieved by forcing a current of air through a small tube inside the ventilator. This produces negative pressure which is used to actively remove air out of the lungs. The “core” working mechanism of the Evone® is presented schematically in Figure 5.

### Surgery

Various surgical upper airway procedures were performed. In general, after the introduction of a standard rigid Kleinsasser laryngoscope (Störz, Tuttlingen, Germany), laryngo-endoscopy using 0, 30, and 70° endoscopes (Störz, Tuttlingen, Germany) was performed in order to assess the pathology. Subsequently, laryngeal microsurgery was carried out using an operating microscope (OpmiVario, Carl Zeiss, Jena, Germany). In most cases, a CO_2_ laser (Lumenis Acupulse Duo, Yokne'am Illit, Israël) was used, either to perform TLM for malignant glottic lesions, laser resections of benign laryngeal lesions, posterior cordotomy in case of posterior glottis stenosis or radial incisions of a subglottic stenosis. When using the laser, the Tritube®'s cuff was covered using a wet cottonoid to protect it from accidental laser damage. Furthermore, the FiO_2_ was reduced below 30% when the laser was in use according to general laser-safety protocols. When subglottic stenosis was treated, additional Mitomycin C application, corticosteroid injection, and balloon dilations were performed.

## Results

**Figure 6:**
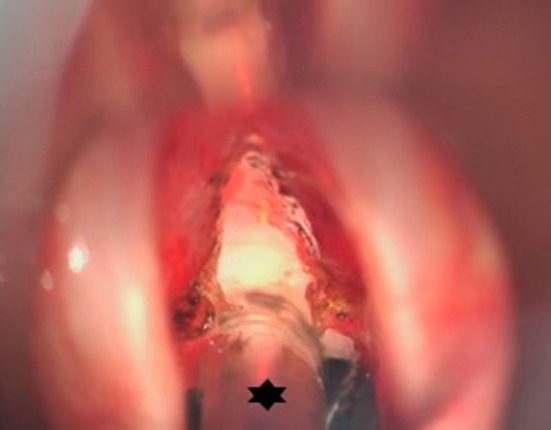
Peroperative microscopic view of the glottis and subglottis after CO2 laser radial incisions in an idiopathic subglottic stenosis (Cotton-Myer grade III) have been performed. Hence the small bore tube (star) allowing minimal traumatic intubation and creating an ideal exposure and working space while maintaining stable ventilation.

**Figure 7:**
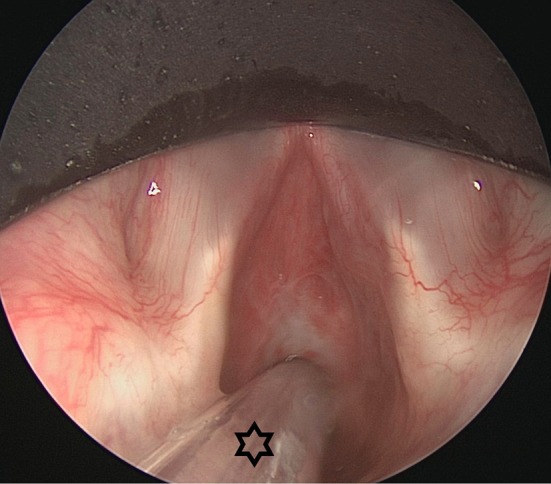
Endoscopic view on an idiopathic subglottic stenosis (Cotton-Myer grade III). The small bore tube (star) allowed for intubation of this severe stenosis. In these severe cases, HFJV could be problematic due to obstruction of the passive air backflow. This problem is tackled in FCV, allowing for good and stable ventilation.

**Figure 8:**
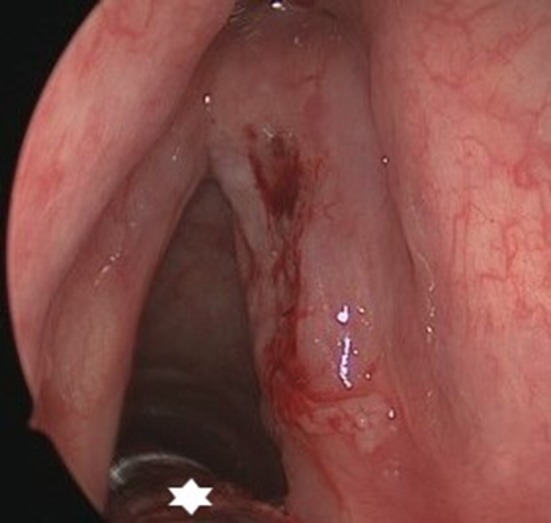
Endoscopic view on a cT2N0 infiltrating squamous cell carcinoma of the right vocal fold with submucosal extension in the sinus of Morgagni. Thanks to the small bore tube (star), margin assessment and achievement of a free posterior margin during TLM are not hindered.

**Figure 9:**
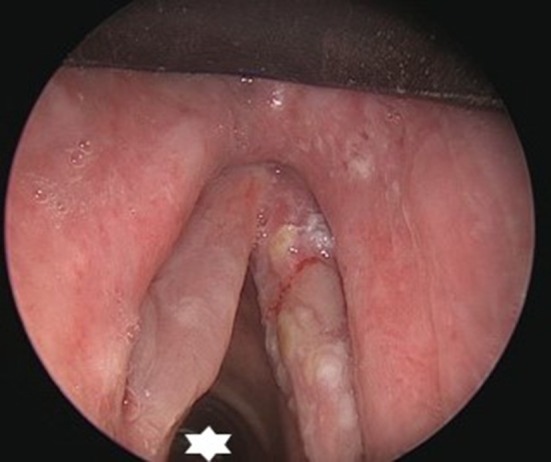
Endoscopic view on a cT1N0 infiltrating squamous cell carcinoma of the right vocal fold. Thanks to the small bore tube (star), an adequate surgical workspace is created.

**Figure 10:**
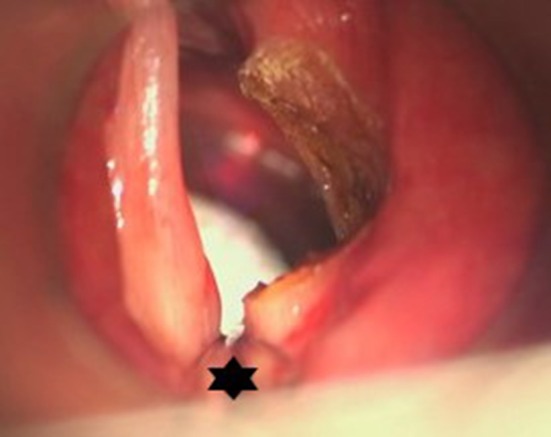
Microscopic view after right-sided posterior cordotomy in a patient with posterior glottic stenosis. The posteriorly located surgical field is not obstructed by the Tritube® (star), and allowed a quick and uncomplicated procedure.

**Figure 11:**
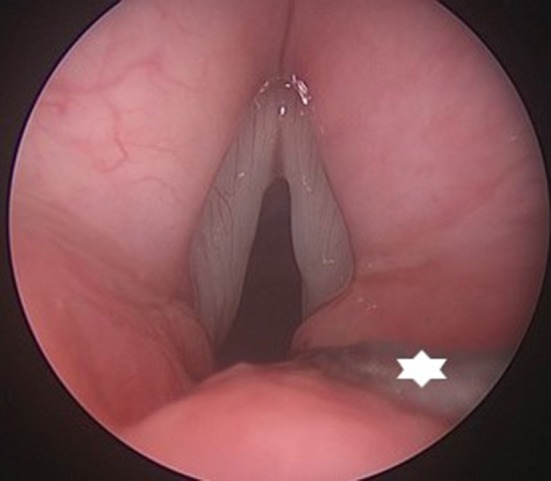
Endoscopic view on a posterior glottic stenosis. The small bore tube (star) provides excellent view on the posterior glottis, without interfering with the evaluation of arytenoid mobility and performance of a posterior cordotomy.

Fifteen consecutive patients were included with a median age of 54 years. In all cases, Evone® FCV ventilation was used during direct laryngoscopy with or without CO_2_-laser. Surgical procedures and indications were laser-assisted endoscopic treatment of idiopathic subglottic stenosis (*n* = 3), tracheal stenosis (*n* = 1), and posterior glottic stenosis (*n* = 2); biopsy and/or TLM for laryngeal (pre)malignancy (*n* = 7) and resection of benign lesions with posterior (supra)glottic location (*n* = 2). The collected data are presented in Table 1. Additional descriptive statistical analysis of these data is shown in Table 2. Mean ventilation duration was 52 min (range 30–115 min, SD 19.6 min), mean surgery duration was 31.7 min (range 15–65 min, SD 13.2 min), mean minimal SaO2 was 96.3% (range 89–100%, SD 4.0%), and mean peak pCO_2_ was 41.4 mmHg (range 31–50 mmHg, SD = 5.5 mmHg). During all the procedures, the small-bore tube allowed a safe ventilation and good visualization of the surgical site, while maintaining stable blood O_2_ and CO_2_ levels. One indication was subglottic stenosis (SGS) (*n* = 3, patients 8, 11, and 13) for which CO_2_- laser radial incisions with balloon dilatations were performed. The small-bore diameter of the Tritube® allowed a safe and minimal traumatic passage past the stenosis and the acquirement of a safe airway, while maintaining a comfortable workspace for the laser-assisted surgery. After completion of the radial laser-incisions, the Tritube® was removed and balloon dilations were performed during apnea. Figures 6, 7 illustrate the use of the Evone® system during treatment of SGS, hence the adequate remaining workspace for the surgeon and favorable exposure. Another indication was the removal, debulking or biopsy-taking of laryngeal tumors (*n* = 7, patients 2, 3, 6, 7, 9, 10, and 12). During these procedures, using the Tritube®, we were able to obtain a safe airway without damaging the tumoral process, avoiding bothersome tumoral bleeds. Furthermore, thanks to the use of the small-bore tube, the surgical site could be visualized in toto (in contrast to a standard endotracheal tube, which, because of its bigger size, often has to be moved during surgery in order to be able to visualize a different part of the surgical site), allowing for superior tumoral margin assessment. During TLM of glottic squamous cell carcinoma (SCC), the small bore tube also allowed for easier resection, especially at the posterior section margin at the level of the arytenoid, which is difficult to address in the presence of a normal bore endotracheal tube, necessitating interruption of the surgical procedure and luxation of the tube into the anterior glottis. Figures 8, 9 illustrate how the Tritube® provides a superior visualization and exposure of the surgical site. Apart from being very helpful during the treatment of subglottic stenosis, the Evone® proved its added value during the endoscopic treatment of other types of airway stenoses (*n* = 3). Patient 4 was treated for a tracheal stenosis and patients 5 and 15 underwent a laser-assisted posterior cordotomy/partial arytenoidectomy for an incapacitating posterior glottic stenosis. Figures 10, 11 illustrate the value of the Tritube® in the treatment (Figure 10) and assessment (Figure 11) of a posterior glottic stenosis.

**Table 1:** Detailed data of included patients.

**Patient**	**Age (yo)**	**Gender**	**Height (cm)**	**Weight (kg)**	**BMI**	**ASA**	**MP**	**Duration of ventilation (min)**	**Duration of surgery (min)**	**Minimal SaO_**2**_ (%)**	**Peak pCO_**2**_ (mmHg)**	**Pathology**	**Surgical procedure**
No. 1	59	F	158	48	19,2	2	2	50	35	100	38	Benign epidermoid cyst on the right aryepiglottic fold	Explorative DL with microsurgery (cold steel)
No. 2	53	M	181	73	22,3	2	2	45	35	100	39	Recurrent cT2N0 squamous cell carcinoma right glottis	DL +Biopsy
No. 3	66	M	172	82	27,7	2	1	50	40	100	40	Bilateral hyperkeratosis of the vocal cords	TLM
No. 4	39	M	175	106	34,6	2	1	60	35	93	40	Iatrogenic tracheal stenosis	DL with CO_2_-laser treatment
No. 5	58	M	182	76	22,9	2	2	40	25	100	40	Posterior glottic stenosis caused by bilateral arytenoid ankylosis (grade IV)	CO_2_-laser assisted posterior cordotomy
No. 6	33	M	190	67	18,6	2	1	40	20	91	36	Neoplasia in the right subglottis	DL + Biopsy
No. 7	54	M	170	72	24,9	3	2	30	15	100	31	Leukoplakia in the right pyriform sinus	TLM
No. 8	43	F	172	68	23,0	1	1	50	30	100	44	Idiopatic subglottic stenosis (Cotton Myer grade III)	CO_2_-laserincision and balloon dilation
No. 9	67	M	186	85	24,6	2	2	115	65	94	38	Chondrosarcoma (grade I) in the right hemilarynx	DL with CO_2_-laser debulking
No. 10	73	M	178	95	30,0	3	2	60	15	89	48	Relapse glottis SCC (with subglottic expansion)	DL + Biopsy
No. 11	54	F	170	96	33,2	2	2	65	50	93	50	Idiopathic subglottic stenosis	CO_2_-laser incision and balloon dilation
No. 12	65	M	172	78	26,4	2	1	40	25	92	37	Suspicious lesion anterior right vocal cord	TLM
No. 13	44	F	169	74	25,9	3	2	45	35	100	48	Subglottic stenosis	CO_2_-laser incision and balloon dilation
No. 14	57	M	186	105	30,4	2	1	45	25	98	43	Recurrent polipoid lesion left vocal process	TLM
No. 15	45	F	160	60	23,4	2	2	45	25	95	49	Posterior glottic stenosis caused by left arytenoid ankylosis (grade III)	Laser –assisted partial arytenoidectomy

*ASA, American Society of Anesthesiologists score; BMI, Body Mass Index; DL, Direct Laryngoscopy; MP, Mallampati Score; yo, years old*.

**Table 2:** Descriptive statistical analysis of ventilation duration, surgery duration, minimal SaO_2_, and peak CO_2_.

***n* = 15**	**Duration of ventilation (min)**	**Duration of surgery (min)**	**Minimal SaO_**2**_ (%)**	**Peak CO_**2**_ (mmHg)**
Mean	52.0	31.7	96.3	41.4
Range	30-115	15–65	89–100	31–50
SD	19.6	13.2	4.0	5.5

Both patients 1 and 14 were admitted for surgery respectively for an inclusion cyst on the right aryepiglottic fold and a recurrence polypoid lesion in the left vocal process. Both masses were successfully removed using cold steel. No complications, adverse events or intra-operative difficulties, were reported during or after any of the 15 procedures.

## Discussion

This observational study is one of the first to report the clinical use of a FCV-device in patients during upper airway surgery. The findings of this study suggest that FCV is a safe and effective method of ventilation as no hypoxia (SaO_2_ <80%), no hypercapnia (pCO_2_ > 50mmHg) and no adverse events (e.g., tube dislocation, obstruction of tube, laser-induced fire of the tube,…) were reported with its use. The combination of the Tritube® with Evone® ventilation also ensured an excellent accessibility and visualization of the surgical site. This is especially true in airway stenosis surgery and TLM for laryngeal cancer. A previous study already demonstrated a significant reduction in positive deep section margins (14% vs. 44%; *p* = 0.04) when using HFJV (which utilizes a small-bore tube with diameter comparable to the Tritube®) compared to standard endotracheal intubation during TLM for Tis-T2 laryngeal cancer. Even though the HFJV was surgically superior compared to standard orotracheal intubation, some anesthesiologic drawbacks, and complications were observed. The most common complications when using HFJV were hypoxia, hypercapnia and emphysema. Moreover, since HFJV relies on an un-cuffed tube, blood aspiration may also represent an issue (14). In our case series, none of the above drawbacks and/or complications were observed using Evone®. This can probably be attributed to the fact that the Evone® utilizes a cuffed tube (whereas the HFJV utilizes an un-cuffed tube) and actively manages the expiratory phase. Besides, in HFJV, the expiratory phase relies completely on the passive backflow of air around the tube. This makes bulky tumors and severe airway stenosis a relative contraindication for the use of HFJV, because they might restrict the backflow around the tube, increasing the risk for hyperinflation. Since the Evone® uses a cuffed tube, and doesn't rely on passive backflow for expiration, bulky tumors and severe stenoses represent no contraindication. Moreover, because of the passive backflow in HFJV, a hindering oscillation of the vocal cords could often be observed. This is also eliminated when using the Evone®. The use of Evone® is also likely to be time saving. Indeed, as mentioned before, using the HFJV often resulted in quick desaturation when oxygen tension was reduced during laser surgery. This sometimes required the surgery to be momentarily halted to allow higher FiO_2_ and resulted in time loss. When using the Evone®, there were no cases of hypoxia that required the surgery to be stopped temporarily. Moreover, in one patient (number 9), the Evone® was able to maintain stable oxygen levels for almost 2 h. Another benefit of using the Evone® with its accompanying small-bore tube is the possibility to obtain a secure airway in patients with an airway stenosis without causing local damage and resulting edema. This potentially decreases the need for a postoperative tracheostomy due to postoperative airway edema in patients with an already compromised airway (e.g., serious edema of the glottis and supraglottic region after radiotherapy). In our opinion and from a surgeon's perspective, Evone® FCV ventilation is indicated for TLM of glottic and supraglottic benign, premalignant, and malignant lesions involving the posterior third portion of the true vocal folds, ventricles, false vocal folds, or aryepiglottic folds and of lesions involving the arytenoids (including vocal process) and the posterior commissure. If these structures would need to be incorporated in a laser-resection to secure adequate margins without actual involvement by malign disease, Evone® is also indicated. In cases of endoscopic airway surgery, Evone® ventilation is the new standard of care in our institution for most glottic, subglottic, and proximal tracheal stenosis cases. Exceptions are the critical subglottic and tracheal stenoses with a lumen diameter of less than 4 mm, as this diameter will not allow the Tritube® to pass. In these cases, alternative ventilation techniques such as supraglottic jet-ventilation or alternative endoscopic surgical techniques such as flexible endoscopic laser-assisted treatment in combination with laryngeal mask ventilation are possible. A last possible indication which we didn't assess as such yet is the ventilation of patients with subglottic or tracheal stenosis undergoing open surgical procedures (cricotracheal resection and segmental tracheal resection). In these cases, the Evone® ventilation will guarantee a minimally traumatic intubation and a safe and stable ventilation while leaving a sufficient workspace for the surgeon to perform (crico)tracheal resection and airway anastomosis. This last possible indication will need further exploration and evaluation.

However, some drawbacks of this new system could be identified. Every time the Evone® is set up, a calibration using an artificial lung is required. It currently takes about 5–10 min to prepare the ventilator. Although this might not be problematic when using the Evone® during elective surgery (where sufficient time for calibration is available), it currently renders the use of the Evone® less obvious in emergency situations. This issue is currently evaluated by the company and an automatic internal calibration-unit will probably soon be released in one of the future versions. Additionally, even though this observational study did not show any clear safety concerns, a recent German study described cases of the Tritube® being blocked by secretions or even becoming dislocated when the patient coughs (3). For these reasons, we strongly advise to administer glycopyrronium preoperatively, to reduce the oral, pharyngeal and tracheal secretions. It is also very important to ensure that the cuff is left deflated until the patient has reached full neuromuscular blockade (TOF = 0). Finally, some concerns remain about the laser safety of the cuff. Even though we did not encounter any incidents, we apply standard safety measures in our center (e.g., FiO_2_ ≤ 30% during use of the CO_2_-laser) and position a wet cottonoid on top of the cuff in order to prevent accidental laser-induced damage of the tube and/or cuff. At this time, the Tritube® is not officially certified as a laser-safe tube, which could be a problem if local or national regulations necessitate formal laser-proof certification of endotracheal tubes used during laser-procedures (TLM of laser-assisted treatment of airway stenosis). Although we never experienced any safety issues related to laser-use in combination with the Tritube® when the strict safety measures mentioned above are taken, the company needs to take this drawback into account, developing a certified laser-safe variant of the Tritube®. From a financial point of view, it is important to note that the Tritube® is significantly more expensive compared to a standard endotracheal tube. In our center; a Tritube® tube is ~70 times more expensive than a standard endotracheal tube. Lastly, some anesthesiologists argued about the slowness of Evone® at implementing changes in parameters (e.g., when the minute volume is changed from 12 to 14 Lmin^−1^). Even though no adverse events due to this delay were reported, this might be troublesome. Moreover, this study has some limitations: due to its retrospective design data are limited and inclusion bias is possible, although consecutive patients were included. Due to the absence of a control group with HFJV or classic endotracheal ventilation, we were not able to prove superiority. Nevertheless, this study was able to demonstrate the safety and efficacy of the Evone® and Tritube® in ENT surgery, using objective parameters.

## Conclusion

The Evone® FCV ventilation system proves to be an excellent way to intubate and ventilate patients undergoing upper airway surgery. It combines excellent accessibility and visibility of the operation site with safe and stable ventilation, tackling many drawbacks associated with classic HFJV.

## Data Availability Statement

All datasets generated for this study are included in the article.

## Ethics Statement

This is a retrospective observational study, performed as a single-center case series in a tertiary setting. Our aim is to elaborate on the technical aspects of working with the Evone® ventilator by Ventinova (Meerenakkerplein 7, 5652 BJ Eindhoven, The Netherlands) in ENT surgery, to assess its efficacy and safety, and to give a critical appraisal of this novel method of ventilation. The study received ethical approval (MP007867) by The Research Ethics Committee UZ/KU Leuven on January 16th, 2019.

## Author Contributions

JM: study set-up, data quality control, data analysis (statistics), drafting manuscript, and review of manuscript. AJ: data collection, data analysis (statistics), and drafting manuscript. KV, JV, and PD: drafting manuscript and review of manuscript. VV: study set-up, drafting manuscript, and review of manuscript.

### Conflict of Interest

The authors declare that the research was conducted in the absence of any commercial or financial relationships that could be construed as a potential conflict of interest.
